# Myofibroblastoma Arising in Mammary Hamartoma: A Case Report

**DOI:** 10.4061/2010/726829

**Published:** 2010-08-01

**Authors:** Diego M. Uchôa, Dênnis Baroni Cruz, Pedro Guilherme Schaefer, Karla Laís Pêgas, Eduardo Cambruzzi

**Affiliations:** ^1^Faculdade de Medicina, Universidade Federal do Rio Grande do Sul, Porto Alegre, Brazil; ^2^Departamento de Patologia, Hospital de Clínicas de Porto Alegre, 2350 Porto Alegre, Brazil; ^3^Faculdade de Medicina, Universidade Luterana do Brasil, Canoas, Brazil

## Abstract

Myofibroblastoma (MFB) is a rare mesenchymal tumor arising in breast's soft tissue with a great variety of microscopic features that can be mistaken with a wide variety of biphasic lesions. The authors report a rare case of myofibroblastoma of the breast arising in a mammary hamartoma (MH), present a review of the clinicopathological features of these lesions, and make some diagnostic considerations. The tumour consisted of a well-circumscribed nodule. MFB component comprised about fifty percent of the lesion and was made up of bipolar spindle cells arranged in fascicular clusters separated by bands of hyalinized collagen. There were fat cells and several residual hamartoma glands intermingled and distorted in MFB area. MFB component was positive for Desmin, CD34, bcl-2, and Calponin. To the best of our knowledge, MFB has not been reported in MH, neither has any of the reports described mammary glands joined within MFB.

## 1. Introduction

 Myofibroblastoma (MFB) is a rare mesenchymal tumor arising in breast's soft tissue [1, 2]. In 1987, Wargotz et al. [2] described a benign tumour of the breast composed of spindle cells arranged in fascicular clusters with interspersed bands of hyalinized collagen and called this lesion “myofibroblastoma”. After that, several cases have subsequently been reported, and it has become clear that MFB of the breast may exhibit a greater variety of morphological features than originally described. Glandular structures have not been described in MFB [3–9]. 

 Mammary hamartomas (MH) comprise about 0.7% of all benign breast masses that were first described in 1928 by Prym [10], who referred to them as “mastomas”. Further cases were reported as adenolipomas and fibroadenolipomas. In 1971, Arrigoni was the first to introduce the term mammary hamartoma, that is further characterized by the variety of mature tissues they contain [11]. 

 The authors report a case of MFB developed in MH. This very rare association may be a potential diagnostic pitfall in the spectrum of biphasic cell lesions of the breast. To our knowledge, the coexistence of MFB and MH in the same mass has not been published.

## 2. Case Report

 A 59-year-old woman presented with a solitary nodule in the left breast which was first noted on routine mammography. Left breast echography showed a sharply demarcated nodule in the breast parenchyma. A complete surgical excision of the mass was performed. 

**Figure 1:**
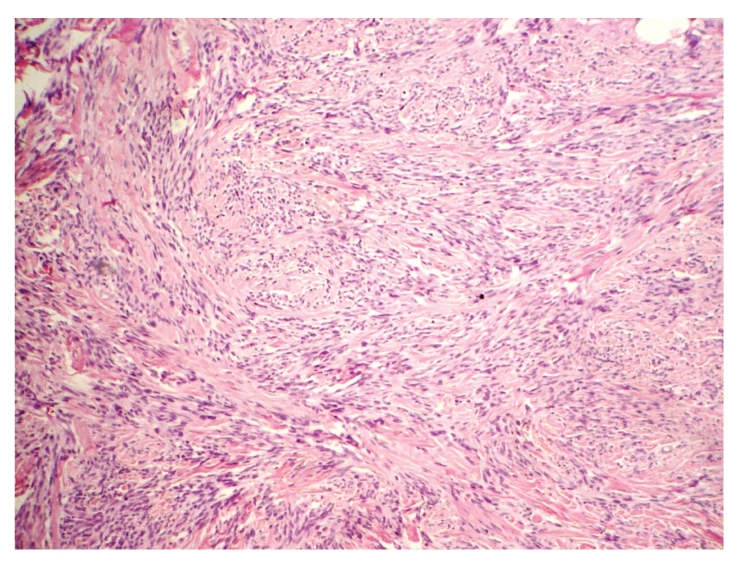
Mammary myofibroblastoma: the lesion shows bundles of slender, uniform, spindle shaped cells typically arranged in clusters and bands of hyalinized collagen distributed throughout the tumor, HE, 100x.

**Figure 2:**
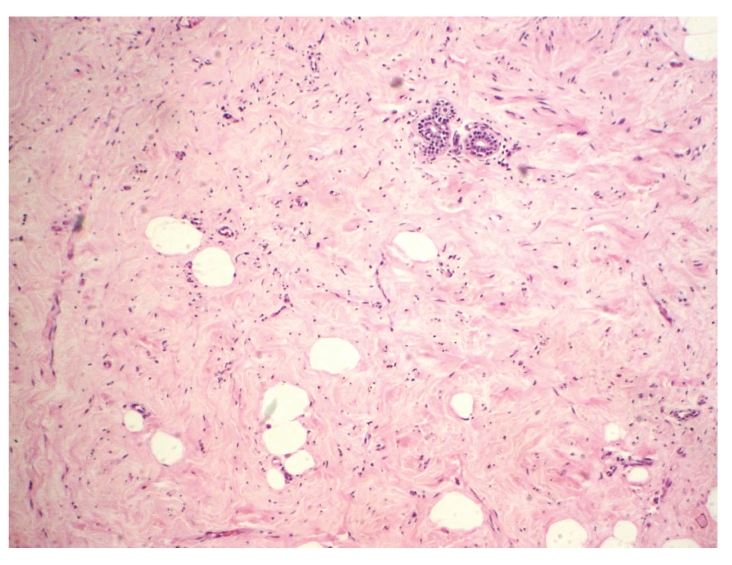
Mammary hamartoma: the process is composed of mammary glandular tissue with a lobular arrangement, fibrous stroma, and adipose tissue in variable proportions, HE, 100x.

**Figure 3:**
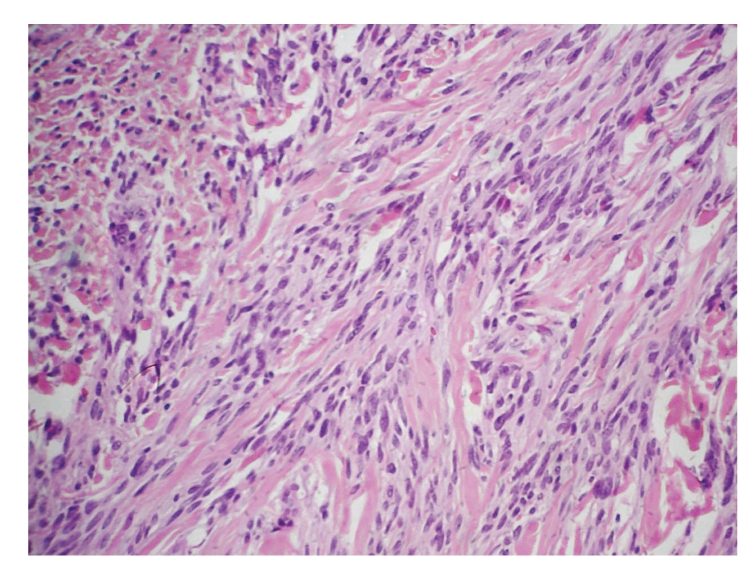
Mammary myofibroblastoma: tumor composed of a homogeneous population of spindle-shaped cells with ovoid nuclei and pale cytoplasm, HE, 400x.

**Figure 4:**
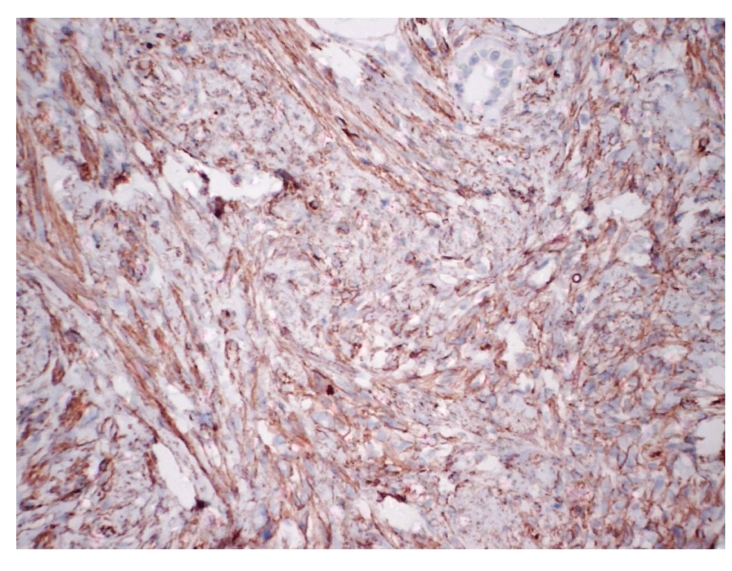
Positive immunoreactivity for CD34 in mammary myofibroblastoma, avidin-biotin technique, 100x.

**Figure 5:**
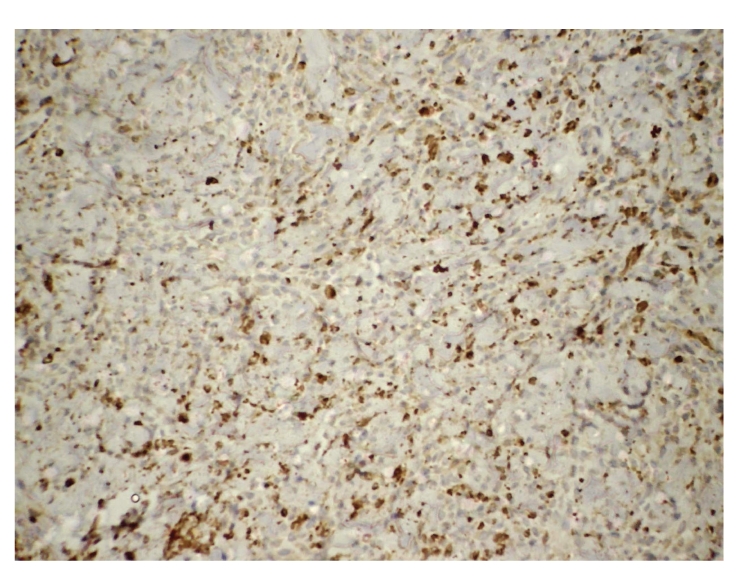
Positive immunoreactivity for calponin in mammary myofibroblastoma, avidin-biotin technique, 100x.

 Gross pathology showed a well-circumscribed, round to slightly lobulated, tan, rubbery, 2,5 cm nodule. On cut sections, a whitish tumour mass with scanty interspersed yellow areas was evident. Microscopically, the tumour had pushing borders and was circumscribed by a pseudocapsule. At scanning magnification, we noticed two distinct tumoral regions. In the “hypercellular area”, classic features of myofibroblastoma of breast were present (Figure 1). The “hypocellular area” revealed a lesion containing scattered epithelial elements embedded in collagenized stroma, typical of mammary hamartoma (Figure 2). The MFB area was composed of bundles of spindle cells with eosinophilic cytoplasm and elongated monomorphic nuclei, adopting a fascicular arrangement and separated by thick collagenous stromal bands (Figure 3). In the stroma, there were scant mature adipocytes, large numbers of mast cells, occasional lymphoid aggregates, and several residual benign-appearing glands. The epithelial elements “entrapped” in the MFB area were not different from the other ones scattered in the MH area. The MFB-MH transitional area was subtle. No mitotic figures, atypical cells, areas of necrosis, smooth muscle, or cartilaginous differentiation were observed. The immunohistochemical profile of MFB component revealed the strong and diffuse staining for Vimentin, Desmin, and CD34 (Figure 4) and focal positivity for Calponin (Figure 5) and bcl-2. Alpha-smooth muscle actin, Actin, S-100 protein, and CD99 and CD10 were negative. The stroma of MH showed diffuse staining for vimentin, and the epithelial elements revealed strong CK7 positivity and focal positivity for bcl-2. Cytokeratin 7 highlighted several residual hamartoma glands admixed to the MFB area. In addition, myoepithelial cells of hamartoma glands were easily identified. There was variable immunoreactivity for Alpha-smooth muscle actin, Calponin, Actin, S-100 protein, and CD10. No recurrence or other complaints have been experienced after a 1-year followup period.

## 3. Discussion

 Primary mesenchymal neoplasms of the breast parenchyma like myofibroblastoma are extremely uncommon, representing less than 1% of mammary neoplasms [1–3]. MFB occurs in the breast of women between 40 and 80 years, referring to a solitary slowly growing nodule. Macroscopically, it is a well-circumscribed encapsulated tumor raging in size from 1,0 cm to 10 cm. Histologically, MFB is an expansile tumor with pushing borders, composed of spindle to oval cells arranged in short, intersecting fascicles, and interrupted by thick bands of collagen. The cells have relatively abundant, ill-defined, eosinophilic cytoplasm with a round to oval nucleus. Necrosis is usually absent. There is a low mitotic index, and it is uncommon entrapment of mammary ducts or lobules within the tumor. Scattered mast cells may be seen in the stroma. The neoplastic cells are usually immunopositive for vimentin, desmin, CD34, and alpha-smooth muscle actin. There is variable immunostaining for bcl-2 protein, CD99, and estrogen and progesterone receptors. Some cases can show lipomatous, smooth muscle, or cartilaginous components [3, 6–9].

 MFB may exhibit a great variety of morphological features, including infiltrating margins and epithelioid component, that can be similar to other spindle-cell benign tumours of the breast, like solitary fibrous tumour (SFT) and spindle cell lipoma (SCL) [2, 3, 7–9]. Mammary MFB and SCL usually have abundant stromal mast cells, and the spindle cells are CD34-positive, but the former is typically positive for desmin. MFB consistently expresses positive muscle immunomarkers in contrary with SFT. Additional evidence of a histologic continuum between MFB, SCL, and SFT has been provided by other reports employing bcl-2, CD10 and CD99 antibodies [3, 6–9]. The MFB spindle-cell area of our tumour expressed bcl2, but not CD10 and CD99 like others reported [3, 6–9].

 MH is further characterized by the presence of a dense hyalinized fibrotic stroma, interlobular fibrosis, variable volume of mature adipose tissue, and pseudoangiomatous hyperplasia [1, 3, 5, 11, 12]. In our paper, the MH area of the breast lump showed all of these features. There are several reports describing primary ductal or lobular breast cancer arising in MH, but neither of these had yet published a fusiform neoplasia arising within MH [1–3, 5, 13, 14].

 Because glandular structures interspersed within MFB are a very rare unpublished finding, some biphasic lesions of the breast, such as adenomyoepithelioma (AME), phyllodes tumor (PT), or even cellular fibroadenoma (CFA), have never been regarded in the differential diagnosis of MFB. So, this unique tumor association (MFB in MH) may be a potential diagnostic pitfall in the spectrum of biphasic cell lesions of the breast [3, 14–19]. The fusiform components of both lesions (CFA and PT) do not show bundles of spindle cells in a fascicular arrangement separated by thick collagen fibers, neither do they have the typical strong and diffuse staining for Desmin and CD34 expressed by MFB spindle cells. Adenomyoepitheliomas usually do not exhibit the thicker bands of hyalinized collagen and are negative for CD34. Entrapped fat cells virtually do not occur in AME too, but mast cells have been found [3, 7, 13, 16, 17, 19]. 

 The differential diagnosis of MFB of the breast can include other reactive processes and benign neoplasms such as nodular fasciitis, fibromatosis, neurofibroma, leiomyoma, stromal sarcoma, malignant fibrous histiocytoma, metaplastic carcinoma, spindle cell liposarcoma, low-grade malignant peripheral nerve sheath tumor, and dermatofibrosarcoma protuberans [2–4, 7, 9, 16, 17]. 

## 4. Conclusion

 Herein, we report a rare case of MFB developed in MH. To the best of our knowledge, this peculiar morphological finding has not been previously reported yet. This very rare association may be a potential diagnostic pitfall in the spectrum of biphasic cell lesions of the breast. The immunohistochemical study of the lesion will be useful. Nevertheless, the simple histologic examination of the entire lesion straightforward turned our diagnosis. In the present nodule, there was a large typical MH area comprising about fifty percent of the mass. Therefore, it was somewhat logical to presume that the entrapped epithelial elements in the MFB component were vestiges of the MH. It could be very difficult to be sure about MFB with entrapped hamartoma epithelial elements without the entire analysis of our tumor.
